# Gastrointestinal Migration of a Pharyngeal Pack During Functional Endoscopic Sinus Surgery (FESS): A Case Report and Preventive Strategies

**DOI:** 10.7759/cureus.19998

**Published:** 2021-11-29

**Authors:** Azza Al-Abri, Hatem Mady, Jawahir Lal, Mohamed Al-Ismaili

**Affiliations:** 1 Anesthesia and Critical Care, Sultan Qaboos University Hospital, Muscat, OMN; 2 Anesthesia and Intensive Care, Sultan Qaboos University Hospital, Muscat, OMN; 3 Gastroenterotolgy Unit, Department of Medicine, Sultan Qaboos University Hospital, Muscat, OMN

**Keywords:** esophago-gastro-duodenoscopy, post-operative nausea and vomiting, post-operative complication, fess, pharyngeal pack

## Abstract

The pharyngeal pack is routinely used in many nasopharyngeal surgeries to reduce the spillage of secretions into the trachea and esophagus. Here we report a case of migration of a pharyngeal pack into the stomach of a patient undergoing functional endoscopic sinus surgery and review risks of delayed recognition and the management of this complication. In this case report, we share our experience to reinforce and highlight the importance of proper documentation of pharyngeal pack insertion and removal to prevent easily avoidable morbidity and mortality. It also highlights the importance of an immediate esophago-gastro-duodenoscopy (OGD) to retrieve the migrated pharyngeal pack as soon as its migration to the gastrointestinal tract is suspected.

## Introduction

Pharyngeal packing is performed in some centers in the world following endotracheal intubation during ear, nose, and throat (ENT) and oral surgeries to reduce perioperative blood ingestion and aspiration. Post-operative nausea and vomiting (PONV) is a common complication following these surgeries which is usually prevented by antiemetics and pharyngeal pack. Also, the pharyngeal pack is aimed to reduce the risk of aspiration [1]. The benefit of the pharyngeal pack in such surgeries is controversial and there is no clear guideline about its use in ENT or facio-maxilary surgeries and it depends on the surgeon's preference [2]. 

## Case presentation

**Figure 1:**
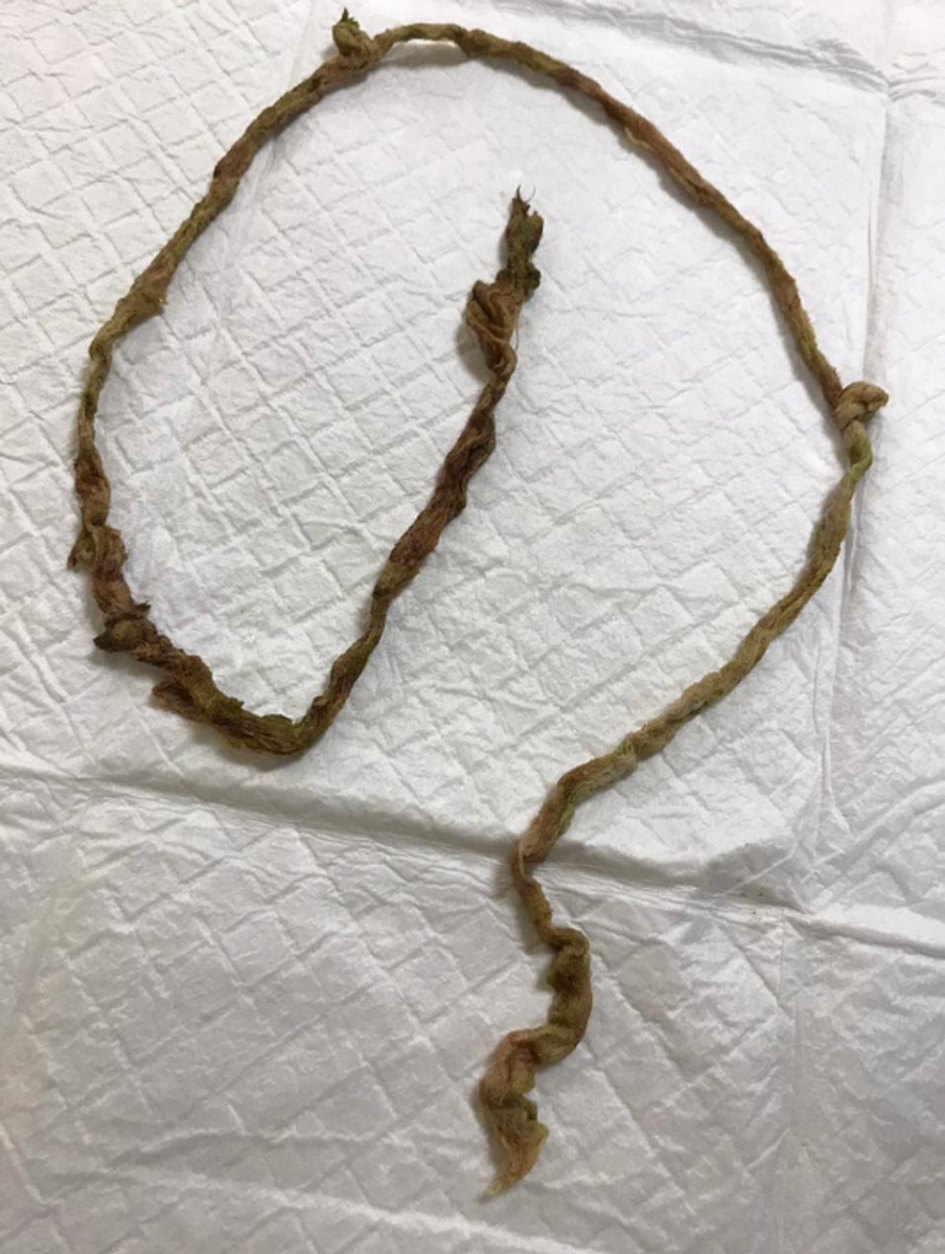
Modified pharyngeal pack made of four pieces of gauze

A 23-year-old woman was diagnosed with severe and extensive fungal sinusitis as a complication of root canal therapy of the left upper molar tooth. She was posted for functional endoscopic sinus surgery (FESS) for management of her extensive fungal infection that caused destructive soft tissue lesions in the left maxillary, ethmoidal and sphenoid sinus with the destruction of the left orbit floor. FESS was done under general anesthesia with a regular endotracheal tube and pharyngeal pack inserted by an anesthesiologist. The regular pharyngeal pack was unavailable, so packing was made using four pieces of unfolded gauze (size 4 by 4 inches) that was tied end to end making a modified pack of 120 cm length as shown in Figure 1.

**Figure 2:**
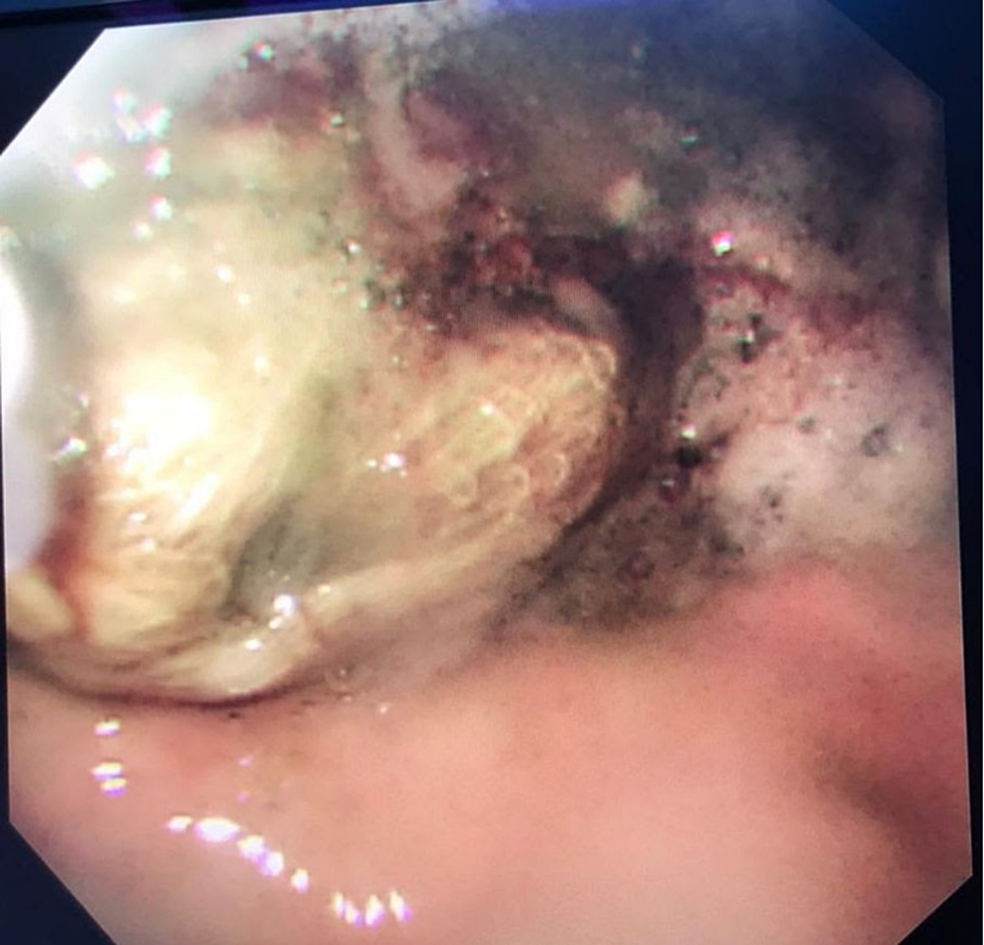
Esophago-gastro-duodenoscopy shows the presence of the migrated pharyngeal pack in the stomach

A documentation of the pharyngeal pack placement and removal was missed during the surgery. Upon the take over of the next anesthesia team , the presence of the pack was not signed over. Before extubation, the pharyngeal pack was checked for by direct laryngoscopy twice, but it was not visualized. Due to lack of documentation, the presence of a pharyngeal pack by the second anesthesia team was missed. The patient was extubated and moved to the post-anesthesia care unit (PACU). After discussion with the first anesthesia team, an alert about insertion of a modified pharyngeal pack was made. An x-ray was deferred as the modified pharyngeal pack lacks a radiopaque line and hence it would not be visualized on a plain radiograph. As the pack was not visualized in the nasopharynx the possibility of migration into the gastrointestinal tract was considered. An emergency esophago-gastro-duodenoscopy (OGD) was performed under sedation and the pack was visualized in the stomach (Figure 2). Pharyngeal pack was extracted eventually endoscopically, and the patient remained clinically stable and had an uneventful recovery after surgery.

## Discussion

The pharyngeal pack is usually inserted in the posterior pharynx in nasopharyngeal, maxillofacial, and dental surgeries after intubation. The pharyngeal pack minimizes spillage of blood and fluids to the trachea and gastrointestinal as well as retains surgical residues like bone and tissue. Missing pharyngeal pack is not an uncommon event with variable methods of diagnosis and retrieval. Failure to visualize the pharyngeal pack in the nasopharynx or oropharynx indicates its migration to either trachea or gastrointestinal tract. Aspiration of the pack can be catastrophic and is reported to cause stridor and respiratory compromise [3]. Swallowing of the pack can result in bowel obstruction. Cases of patients vomiting and then swallowing of pharyngeal pack post-extubation were reported [4].

There are multiple studies on the effect of pharyngeal packs in reducing PONV and sore throat. In septorhinoplasty where induced hypotension is used, the use of a pharyngeal pack is not recommended due to lack of benefit on PONV and increased risk of complications including its retention [5,6]. A recent systematic review of 13 papers concluded that there is no indication for the routine use of pharyngeal packs in ENT, maxillofacial and dental procedures [7]. One randomized control study recommends against the use of routine pharyngeal pack specifically in FESS due to a higher incidence of throat pain 24 hours post-surgery in pharyngeal pack group compared to no pharyngeal pack and no difference in PONV at four hours and 24 hours [8]. A recent study comparing pharyngeal pack with no pharyngeal pack based on a preoperative and post-operative gastric volume using ultrasound found a significantly higher gastric volume and incidence of PONV in the no pharyngeal pack group. Higher gastric volume is due to a higher perioperative ingested blood volume, which can be associated with a higher perioperative pulmonary aspiration [9].

Different techniques are advised to secure the throat pack such as fixing the end of the pharyngeal pack either to endotracheal tube fixation or to the patient's face by a piece of tape so it is visible outside to the surgeon and the anesthetist. Clear documentation of the presence of pack including insertion time of pharyngeal pack on the whiteboard to be followed by the removal time.

This case was presented in anesthesia mortality and morbidity rounds and a protocol for the use of a pharyngeal pack is being established. It is recommended to have clear and visible documentation of the pharyngeal pack in and out time on the operating room whiteboard by the circulating nurse. Also, a new section for pharyngeal pack insertion in the electronic anesthesia chart system was added to prevent recurrence of such complications in the future.

## Conclusions

The routine use of pharyngeal packs in ENT surgeries is still controversial as studies report conflicting data about its benefit in reducing sore throat and post-operative nausea and vomiting (PONV). Migration of the pack may lead to serious complications including airways and bowel obstruction. Proper documentation, clear signs over communication and routine preventive measure are paramount to prevent such complications. If indicated, the use of a radiopaque gauze pack (Raytec gauze) is recommended. Early recognition of missing pharyngeal pack and endoscopy is essential to prevent further migration into a gastrointestinal tract and bowel obstruction.
